# A High Dose, Not Low Dose, of Vitamin D Ameliorates Insulin Resistance in Saudi Women

**DOI:** 10.3390/jcm11216577

**Published:** 2022-11-06

**Authors:** Shareefa AlGhamdi, Hanan AlHarthi, Sawsan Khoja, Amin AlJefri, Huda F. AlShaibi

**Affiliations:** 1 Department of Biochemistry, Faculty of Sciences, King Abdulaziz University, Jeddah 23623, Saudi Arabia; 2 Vitamin D Pharmacogenomics Research Group, King Abdulaziz University, Jeddah 23623, Saudi Arabia; 3 Experimental Biochemistry Unit, King Fahd Medical Research Center, King Abdulaziz University, Jeddah 23623, Saudi Arabia; 4 Division of Family Medicine, Department of Internal Medicine, King Abdulaziz University Hospital, Jeddah 23623, Saudi Arabia; 5 Embryonic Stem Cell Unit, King Fahd Medical Research Center, King Abdulaziz University, Jeddah 23623, Saudi Arabia

**Keywords:** vitamin D deficiency, insulin resistance, insulin, type 2 diabetes, body mass index, 25(OH)D3

## Abstract

Vitamin D has been traditionally seen to be mainly involved in the regulation of bone homeostasis. However, vitamin D has also been clinically linked to various diseases, including metabolic syndrome. The aim of this study was to examine the effect of low and high doses of a vitamin D supplement on the serum levels of 25(OH)D3 and insulin resistance. A total of 120 females were recruited in this study and supplemented weekly with 25,000 IU vitamin D or 50,000 IU vitamin D for three months. Anthropometric measurements were taken at the beginning of the study. Blood samples were collected at the beginning of the study to determine the baseline of the clinical variables and collected again after three months. Insulin resistance was measured using Homeostatic Model Assessment for Insulin Resistance (HOMA-IR). After vitamin D supplementation, a non-significant increase was observed in the serum levels of 25(OH)D3 in the group treated with a low dose of vitamin D (LDVD) and a highly significant increase was seen in the group treated with a high dose of vitamin D (HDVD). In the group treated with a higher dose (HDVD), a significant improvement in insulin sensitivity was observed. The high dose of vitamin D (50,000 IU) supplementation was more effective in both correcting the blood levels of vitamin D and improving the sensitivity of insulin.

## 1. Introduction

Vitamin D has extremely important functions in the body as it functions as a hormone; thus, it affects various systems in the body. Vitamin D deficiency is one of the key public health issues globally. It is estimated that about 1 billion people worldwide have low blood levels of vitamin D [1]. Vitamin D acts more like a hormone rather than a nutrient and our bodies have a special ability to synthesize vitamin D from sunlight, unlike other vitamins. Its main function is regulating and improving the capacity to absorb calcium to avoid the risk of bone fractures [2]. As vitamin D deficiency is an independent risk factor for total mortality in the general population, the high prevalence of vitamin D deficiency is a major public health concern [3,4]. The link between vitamin D deficiency and its negative health consequences was discovered during the early industrialization of northern Europe in the late 19th century. At that time, coal burning and other modern developments limited sun exposure for children, which resulted in rickets, a bone-deforming disease [5]. Vitamin D deficiency is linked mainly to rickets in children and osteoporosis in adults. Over the course of the 20th century, this issue was addressed in various ways; in addition to an adequate amount of sunshine wherever possible, a nutritional approach that focused on a well-balanced diet has been helpful for several generations of children, including the fortification of milk and cereal products and vitamin D supplements, among other proactive measures to ensure that vitamin D deficiencies would be minimal. Consequently, rickets is not a major issue in current times. In addition to the effect of vitamin D deficiency on bones, many studies have linked vitamin D deficiency to various health issues including obesity, cardiovascular diseases, cancer, diabetes, and mental disorders [6,7,8,9].

Among metabolic disorders, vitamin D deficiency has been linked to obesity, insulin resistance, and diabetes [10,11,12]. Insulin resistance is a condition where some people struggle to manage their blood sugar levels. When this condition occurs, the body’s tissues are unable to use blood sugar effectively and they also have difficulty recognizing when the levels of sugar in their bloodstream rise. This leads to high glucose and insulin levels, which can cause weight gain, fatigue, and mood swings, among other symptoms.

In several cross-sectional studies, a low blood level of vitamin D was found to be associated with a higher incidence of obesity [13], hyperglycemia [14], diabetes [15], and insulin resistance [16]. Intervention studies have not adequately identified or observed correlations between vitamin D deficiency and poor glucose tolerance and diabetes in humans. If vitamin D treatment improves glucose metabolism, these benefits are most likely to be seen in patients who have low levels of the vitamin, and the results therefore are inconclusive [17,18,19].

In Saudi Arabia, the rising prevalence of obesity and diabetes is a serious public health problem of the 21st century and it is increasing at an alarming rate [20,21,22]. On the other hand, the prevalence of vitamin D deficiency in the Saudi population is very high; in the western region, for example, it has been estimated to be 80% [23], and in other regions has reached approximately 50% in students and 44% in employees, indicating a serious health concern that needs an effective awareness program [24].

The aim of the present study was to investigate the effect of two different doses (a high and a low dose) of vitamin D on both insulin resistance and the level of serum vitamin D in Saudi women who do not have diabetes, and then examining how normalizing the level of vitamin D could affect insulin resistance. If correcting vitamin D deficiency improves insulin sensitivity, it may slow the progression of type 2 diabetes and metabolic syndrome, which includes insulin resistance as a key component.

## 2. Materials and Methods

### 2.1. Study Design

The current study was approved by the Unit of Biomedical Ethics at King Abdulaziz University. All experiments were performed in accordance with relevant guidelines and regulations. All participants signed a consent form indicating their informed consent and held the right to drop out of the study at any time. A cohort of 120 women aged 18–54 years was recruited from the local community in Jeddah, Saudi Arabia, by local advertisements. This study was carried out between May 2017 and January 2018 (to cover a mix of seasons of the year) as an intervention prospective cohort study. The study involved experiments that use a within-subjects design, so no control group was included. The health status of all participants was verified by a physician at the family medicine department in King Abdulaziz University Hospital (KAUH). The eligible participants were then blindly divided into two groups, each comprising 60 participants. According to the questionnaire, the most commonly prescribed dose of vitamin D is 50,000 IU weekly. After normalizing the serum level of 25(OH)D3, a lower dose is usually prescribed (25,000 IU weekly) to maintain the serum level of 25(OH)D3. Thus, these two doses of vitamin D were selected. The first group was assigned as the low-dose vitamin D (LDVD) group, treated with 25,000 IU/week. The second group was supplemented with a high dose of vitamin D (HDVD) (50,000 IU/week) and assigned as the high-dose vitamin D (HDVD) group. At the beginning of the study, baseline fasting blood samples were collected to measure the biochemical variables of each group and to compare between the LDVD and HDVD groups. The participants from each group were asked to orally take one tablet of vitamin D3 for 3 months (one tablet of 25,000 IU or 50,000 IU per week). The vitamin D3 serum levels (25 OH vitamin D) and other biochemical markers were examined again at the end of the study.

### 2.2. Exclusion Criteria

Participants who were unable to provide informed consent were excluded. Women who were pregnant, lactating, or going through the menopause were also excluded from the study. The exclusion criteria also included regular use of vitamin D supplements, more than 10 micrograms (400 IU) vitamin D daily, a history of chronic and acute diseases, prescribed calcium supplements, parathyroid hormone (PTH), and history of diseases that might limit their ability to participate in the study (physically or mentally) and/or affect the results, including medications disturbing vitamin D and glucose metabolism.

### 2.3. Anthropometric Data

All of the anthropometric data and lifestyle characteristics of all participants were collected from the questionnaire through a personal interview. The demographic and anthropometric data were collected at the beginning of the study before vitamin D supplementation. The main collected data included age, marital status, level of education, past medical history, smoking, physical activity, vitamin D and calcium supplementation, use of pharmaceutical drugs, dairy consumption, sun exposure, clothing case, and sunblock usage. The subjects were weighed (while wearing minimal clothing) on an electric scale (kg), height without shoes was measured (cm), and waist was measured (cm). The body mass index (BMI) was calculated using weight in kilograms divided by height in meters squared. The body fat percentage (BFP) was measured by an electric scale. The participants’ blood pressure and heart rate were also recorded.

### 2.4. Biochemical Analysis

All materials and reagents used in the laboratory experiments of this study were of analytical grade. The laboratory work was carried out in the Chemistry Lab at KAUH. In brief, after overnight fasting for at least 8 h, blood samples were collected, and the serum was prepared. Serum vitamin D [25(OH)D3], PTH, calcium (Ca), magnesium (Mg), phosphorus (P), lipid profile (triacylglycerol (TAG), low-density lipoprotein (LDL), high-density lipoprotein (HDL), and cholesterol (CHOL)), albumin, creatinine, C-reactive protein (CRP), glucose, and insulin were all measured using fully automated systems at KAUH. All measurements were repeated at the end of the study after the completion of three months of supplementation with either the low or high doses of vitamin D. Insulin resistance were measured using HOMA-IR according to the equation:

HOMA-IR = fasting glucose (mmol/L) × fasting insulin (µIU/mL)/22.5

The normal HOMA-IR value for a healthy human ranges from 1.7 to 2.0, with significant insulin resistance being >2.9 [25].

### 2.5. Statistical Analysis

The data obtained during the study were analyzed utilizing IBM SPSS Statistics for Windows, version 23 (IBM SPSS, IBM Corp., Armonk, NY, USA). Graphs were generated by GraphPad Prism, version 8.0. A Shapiro–Wilk test was utilized to evaluate normal value distribution. The data were expressed as mean +/- standard error of the mean (minimum–maximum). The significance between LDVD and HDVD was conducted using a two-way ANOVA followed by Tukey’s multiple comparison test for repeated measures. The difference was considered significant if the *p*-value < 0.05.

## 3. Results

### 3.1. Demographic and Anthropometric Data

The demographic and anthropometric data of all participants were analyzed (Table 1). Age and BFP data were not normally distributed, thus, the minimum and maximum are presented. None of the participants were smokers. All participants were of Saudi nationality (Arab). The education level of all participants varied between high school (20%, n = 24), bachelor (69.2, n = 83), and postgraduate (10.8%, n = 13).

### 3.2. Veil Style and Sun Exposure

All of the women in this study wore a headscarf, given the environment and religious culture in society. However, the veil level was different: 5.1% wore a full-body veil covering the eyes and hands, while 85.7% wore a full-body veil with a face covering, but without eyes and hands being covered. This group formed the majority of the participants. Approximately 9.2% wore a full-body veil, but without covering the face or hands.

**Figure 1:**
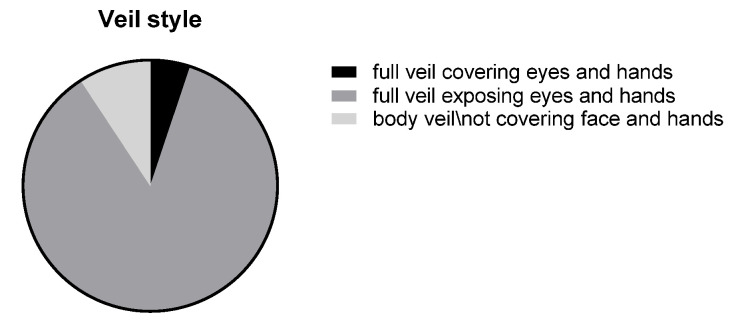

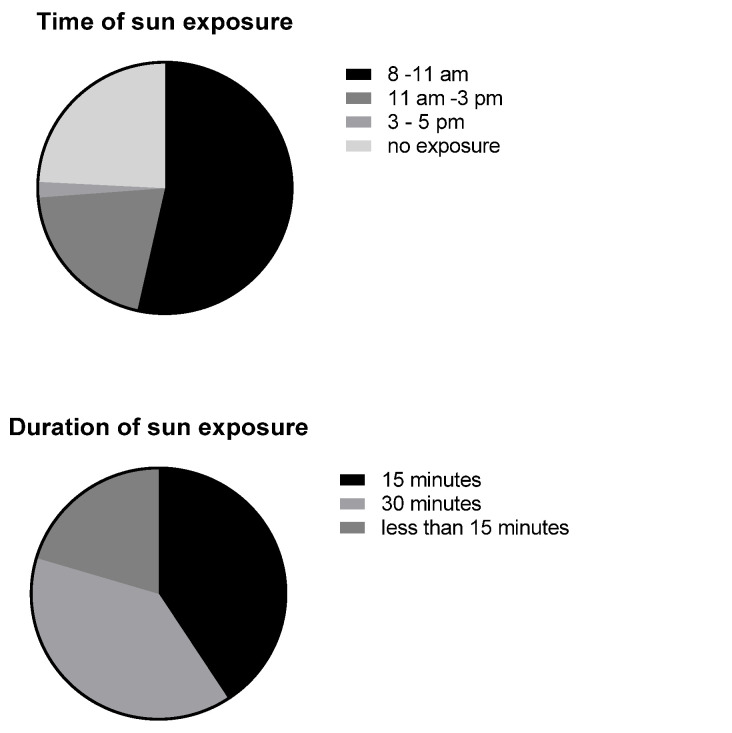
Veil style, duration, and time of sun exposure for all participants.

According to the questionnaire, there was a variation in the time of exposure to the sun. Most of the participants (53.3%) had exposure to the sun from 8 to 11 a.m., while 20.3% and 2% had exposure to the sun from 11 a.m. to 3 p.m. and from 3 to 5 p.m., respectively. About 24.2% were never intentionally exposed to the sun. There was also a difference in the duration of sun exposure per day. About 40.8% usually had exposure to the sun for a quarter of an hour and about 38.8% for half an hour. The rest of the participants usually had exposure for less than a quarter of an hour (Figure 1).

### 3.3. Biochemical Measurements

To assess the health status of the participants, the baseline levels of lipid profile, Mg, P, PTH, albumin, CRP, and creatinine were measured at the beginning of the study. After 3 months of vitamin D supplementation with a low or high dose, the serum levels of these markers were measured again (Table 2). The results showed that they were all within normal levels and no significant change was observed after vitamin D supplementation, except for high-sensitivity CRP, which was significantly decreased in both the LDVD and HDVD groups.

### 3.4. Vitamin D, PTH, and Calcium

**Figure 2:**
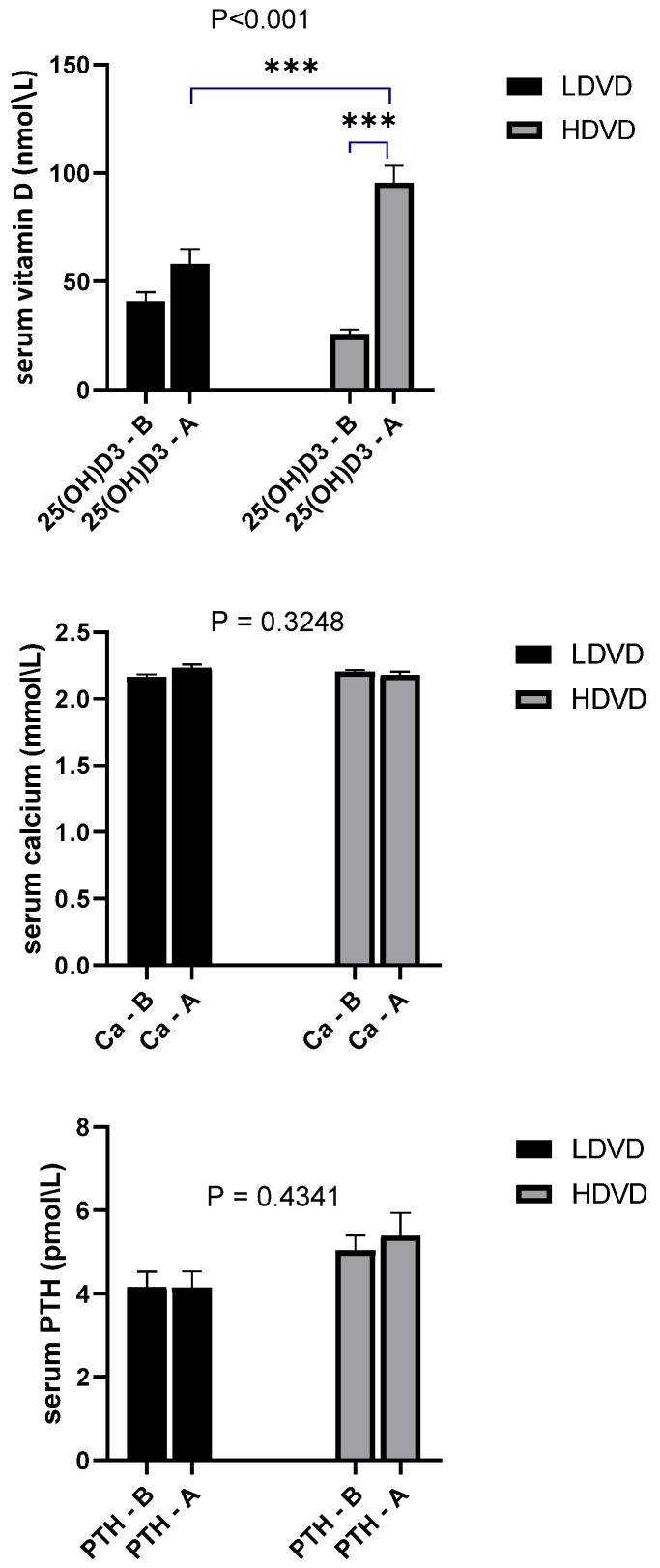
Comparison of fasting serum levels of 25(OH)D3, Ca, and PTH. Comparison was carried out between LDVD and HDVD groups and their levels before (B) and after (A) treatment of the same group using two-way analysis of variance followed by Tukey’s multiple comparison test. Bars represent the serum concentration of biochemical variable. Error bars represent standard error of the mean (SEM). Exact *p*-values are recorded on graphs. Data were considered significant if *p* < 0.05. *** *p* < 0.001.

The biochemical measurements of serum 25(OH)D3, PTH, and Ca were measured at the beginning of the study to determine the baseline levels (Figure 2). No significant difference was observed in serum 25(OH)D3 baseline concentrations between the LDVD and HDVD groups. All groups were characterized by low serum levels of 25(OH)D3, showing that the participants had vitamin D insufficiency/deficiency (<50 nmol/L). After 3 months in the LDVD group, no significant difference was observed before vitamin D supplementation (40.48 ± 4.39 nmol/L) and after supplementation (58.10 ± 6.53 nmol/L). In the HDVD group, a highly significant difference was observed before vitamin D supplementation (32.15 ± 2.85 nmol/L) and after supplementation (100.02 ± 7.24 nmol/L) (*p* < 0.0001). Moreover, a significantly higher 25(OH)D3 level was seen in the HDVD compared to the LDVD group (*p* < 0.01). The PTH and Ca levels did not change significantly in either the LDVD group or HDVD group after 3 months of vitamin D supplementation, and no significant changes were observed when the HDVD group was compared to the LDVD group.

### 3.5. Glucose, Insulin, and HOMA-IR

**Figure 3:**
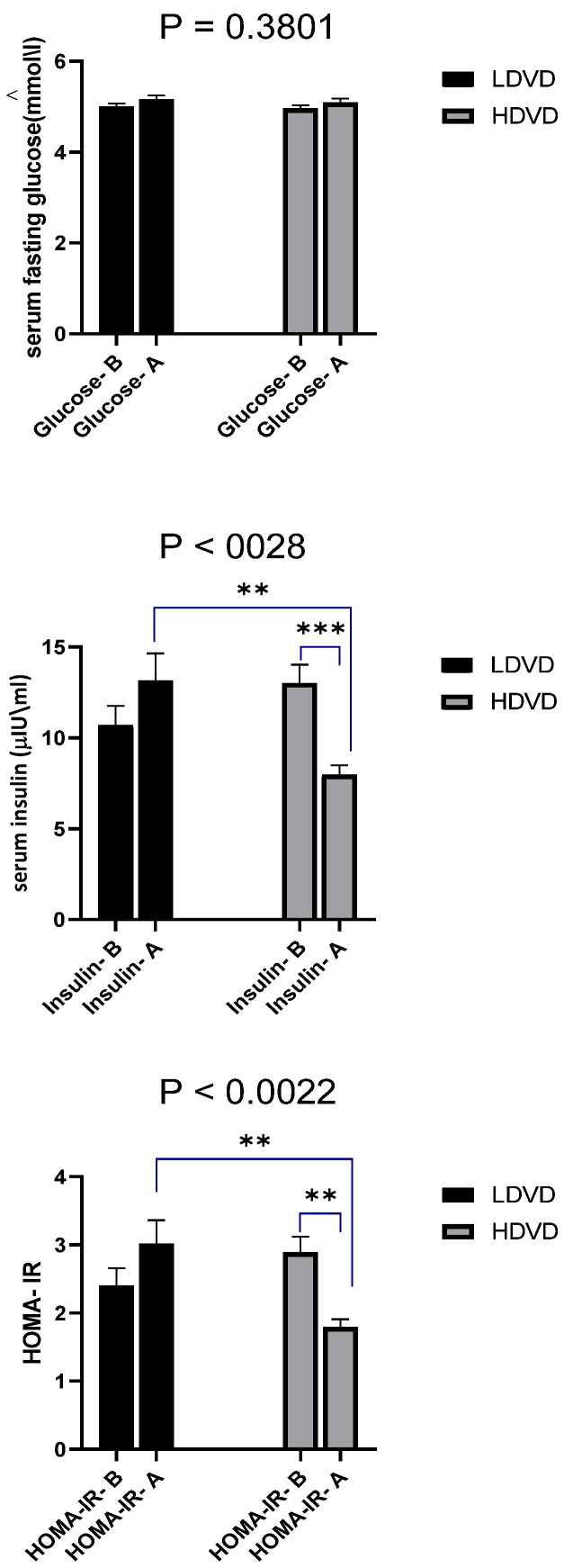
Comparison of measurements of glucose, insulin and HOMA-IR. Comparison was carried out between LDVD and HDVD groups and their levels before (B) and after (A) treatment of the same group using two-way analysis of variance followed by Tukey’s multiple comparison test. Bars represent the serum concentration of the biochemical variable. Error bars represent standard error of the mean (SEM). Exact *p*-values are recorded on graphs. Data were considered significant if *p* < 0.05. ** *p* < 0.01, *** *p* < 0.001.

The fasting levels of glucose and insulin as well as HOMA-IR (Figure 3) showed there were no significant differences observed in the serum fasting glucose levels (LDVD: 5.00–5.02 nmol/L) (HDVD: 4.96–5.08 nmol/L), confirming that all participants were non-diabetics and thus had normal blood glucose levels. In the LDVD group, no significant differences were observed in either the insulin levels (B: 10.7, A: 13.6 µIU/mL) or HOMA-IR (B: 2.89 ± 0.23, A: 1.79 ± 0.11) after 3 months of supplementation compared to the baseline levels. In the HDVD group, the mean values of HOMA-IR before and after treatment were significantly decreased (B: 2.89 ± 0.23, A: 1.79 ± 0.11). In addition, a significant decrease in serum insulin levels (B: 13.02, A: 7.96 µIU/mL) was observed. A significant decrease in both serum insulin levels and insulin resistance measured by HOMA-IR was observed in the group treated with HDVD when compared to the LDVD group.

**Figure 4:**
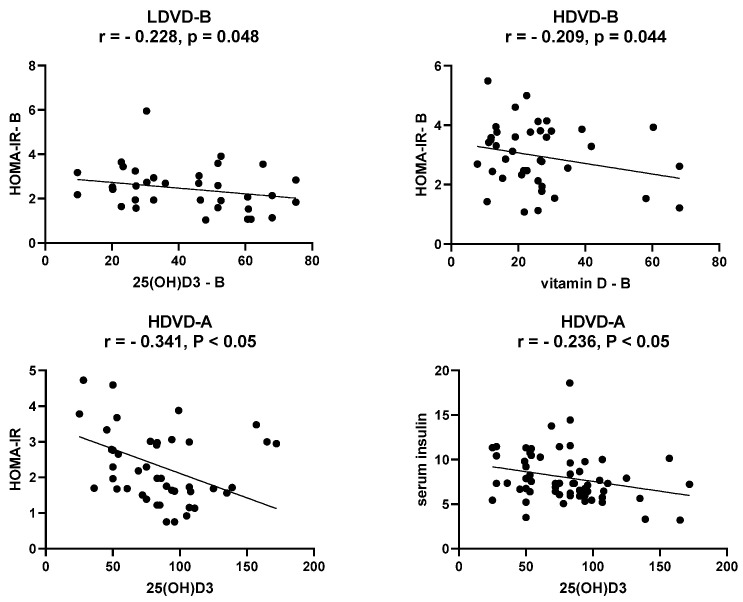
Inverse correlation between serum 25(OH)D3, insulin, and HOMA-IR in LDVD and HDVD group before (B) and after (A) three months of vitamin D supplementation (50,000 IU).

Pearson analyses (Figure 4) were performed to assess the relationships between the serum 25(OH)D3 level, serum insulin level, and insulin resistance degree measured by HOMA-IR. At the baseline levels, serum 25(OH)D3 was oppositely correlated only with HOMA-IR in both the LDVD and HDVD groups (r = −0.228, *p* = 0.048, r = −0.209, *p* = 0.044, respectively). No correlation was observed between 25(OH)D3 and either insulin or glucose levels. After vitamin D treatment, the results showed no significant correlation between 25(OH)D3, insulin level, and HOMA-IR in the LDVD group. However, a significant opposite correlation (Table 3) was observed between vitamin D and insulin level (r = −0.236, *p* < 0.05) and HOMA-IR (r = −0.341, *p* < 0.05) in the HDVD group.

## 4. Discussion

Vitamin D deficiency is a global health issue [26]. Vitamin D deficiency has long been linked to various skeletal disorders and in recent decades has been linked to other diseases including diabetes, obesity, and insulin resistance [5]. Obesity is the major leading cause of insulin resistance [27]. The aim of the present study was to assess the effect of two different doses of vitamin D, that are most commonly prescribed, on the insulin resistance of Saudi women.

This study identified several demographic and lifestyle factors related to vitamin D levels. Studies have stated that vitamin D intake, age, BMI, blood pressure, and body fat levels are the main determinants of serum 25(OH)2 D3 concentration. The participants in this study were Saudi females and, according to the data analysis, most participants (82.5%) were characterized by a BMI higher than 25, indicating that the majority of the participants were overweight or obese. According to the results of a recently published study, the prevalence of obesity in the Saudi population is 24.7% [28]. It is worth noting that the weight of most of the participants was not considered to be very high; however, their mean height ranged between 156 and 158 cm, explaining the increase seen in their BMI. Therefore, it is recommended to inform females in this population to use BMI rather than absolute weight when monitoring and assessing their degree of being overweight or obese.

The present study showed that the participants were characterized by vitamin D deficiency, where 76% had a serum vitamin D level less than 50 nmol/L. These results are in accordance with many previous studies that showed a prevalent incidence of vitamin D deficiency in the Saudi population of different age and gender groups [29], and the incidence is more prominent in females in the Saudi population [30,31]. The results also showed that most participants wore veils and that the time and duration of sun exposure was inadequate. According to Alshahrani et al. (2013), the peak time for vitamin D production is from 10:00 a.m. until 2.00 p.m. [32]. Thus, the vitamin D insufficiency/deficiency seen in this study might be attributed to several factors such as low vitamin D intake, wearing veils, and the time and duration of sun exposure. Therefore, it is recommended to advise veiled women to maintain taking vitamin D supplementation to avoid osteoporosis. About 40.8% of the participants indicated that they were only exposed to the sun for about 15 min per day. The results also showed that supplementation with the higher dose of vitamin D (50,000 IU) was more beneficial than the lower dose (25,000 IU) in correcting the level of serum 25(OH)D3. Thus, it is recommended for Saudi females to use a higher dose of vitamin D to achieve and maintain the optimal level of vitamin D, as well as increasing their exposure time to the sun. Fortified foods and reasonable dietary supplements, as well as sensible sun exposure, are recommended to achieve an acceptable range of vitamin D in the general population and to prevent vitamin D deficiency.

The current results showed that treatment with 50,000 IU of vitamin D for three months increased the sensitivity to insulin levels and significantly reduced the insulin resistance index measured by HOMA-IR. In the LDVD group, a slight non-significant increase was seen in the circulating insulin level, which agrees with a recent study conducted by Rafiq and Jeppesen [33]. It has been stated that vitamin D stimulates insulin secretion in the beta cells of the pancreas and improves insulin sensitivity in target cells, i.e., muscle, adipose tissue, and liver [34,35]. Low blood levels of 25(OH)D3 have been linked to hyperglycemia and insulin resistance [33,36]. Therefore, high doses of vitamin D and not low doses can be implicated positively in ameliorating the insulin sensitivity in overweight people. One assumption could be that the serum level of 25 (OH) vitamin D3 needs to reach a certain blood concentration to affect insulin sensitivity. It is known that insulin secretion is affected by PTH, which is mediated by raising the concentration of Ca [37,38]. In the present study, the HDVD group was characterized by non-significantly higher levels of PTH, which is known to increase Ca reabsorption. Consequently, it is also possible that the increased serum level of 25(OH)D3 affects the absorption of Ca, which is then regulating insulin secretion. Our findings are supported by previous studies [39]. We also found a significant negative correlation between serum 25(OH)D3 and both insulin resistance and HOMA-IR. These findings are in agreement with other clinical studies [40,41]. Vitamin D function is mediated by its receptors [42]. Pancreatic beta cells have the receptors for vitamin D [43], which suggests a role of vitamin D in the functions of the beta cells. Previous studies have demonstrated that knocking out vitamin D receptors to cause vitamin D deficiency resulted in impaired insulin secretion induced by glucose [10,44]. It has also been found that vitamin D can directly increase insulin receptor expression, thereby increasing insulin stimulation of glucose transport [45]. Several mechanisms could contribute to the effect of vitamin D on insulin resistance; however, the exact underlying mechanism is still unclear. Therefore, further research is needed to determine this mechanism. Altogether, our results suggest that correcting the serum level of 25(OH)D3 might be particularly important in ameliorating insulin resistance, thus reducing the risk of developing diabetes mellitus in overweight/obese populations.

## 5. Conclusions

Supplementation with the higher dose of vitamin D (50,000 IU) had a significant effect in improving the serum levels of vitamin D. Supplementation with the high dose of vitamin D ameliorated insulin resistance and had a positive effect on improving insulin sensitivity in overweight females. Therefore, a high dose of vitamin D is highly recommended for females in the Saudi population who are characterized by vitamin D insufficiency/deficiency.

## 6. Limitations

The main limitation of this study was the small sample size, which might affect the outcome of some statistical analyses.

## Figures and Tables

**Table 1:** Anthropometric and demographic data of the study groups.

	LDVD (n = 60)	HDVD (n = 60)	
Nationality	Arab (Saudi) 100%	Arab (Saudi) 100%	NS
Age (years)	33.20 ± 2.56 (20–54)	26.18 ± 1.62 (18–49)	NS
Weight (kg)	70.68 ± 3.55	67.37 ± 2.49	*p* < 0.05
Height (cm)	156.7 ± 1.23	158.1 ± 1.19	NS
BMI	28.57 ± 1.49	26.81 ± 1.45	NS
Waist circumference (cm)	85.66 ± 2.99	82.51 ± 2.01	*p* < 0.05
Body fat percentage (BF%)	32.66 ± 1.72 (14.60–44.70)	29.36 ± 1.23 (13.50–39.0)	*p* < 0.05
Systolic blood pressure (SBP) (mmHg)	119.1 ± 2.47	119.3 ± 2.72	NS
Diastolic blood pressure (DBP) (mmHg)	73.04 ± 2.04	72.32 ± 2.91	NS
Heart rate (HR)	81.61 ± 3.01	84.79 ± 2.39	NS
Smoking	0%	0%	NS

NS = non-significant.

**Table 2:** Biochemical measurements.

	LDVD-B	LDVD-A	HDVD-B	HDVD-A	
CA (mmol\L)	2.164 ± 0.02	2.23 ± 0.03	2.224 ± 0.013	2.181 ± 0.02	NS
Albumin (g\L)	36.0 ± 0.77	36.26 ± 0.89	38.75 ± 0.58	38.74 ± 0.64	*p* < 0.05 ^c^
P (mmol\L)	1.042 ± 0.03	1.03 ± 0.0.32	1.03 ± 0.03	1.05 ± 0.02	NS
PTH (pmol\L)	4.16 ± 0.40	4.15 ± 0.35	5.03 ± 0.37	5.39 ± 0.55	NS
MG (mmol\L)	0.873 ± 0.02	0.875 ± 0.02	0.853 ± 0.01	0.839 ± 0.01	NS
TAG (mmol\L)	0.933 ± 0.12	0.923 ± 0.08	0.745 ± 0.06 (0.39–1.18)	0.7175 ± 0.05 (0.3–1.35)	NS
HDL (mmol\L)	1.628 ± 0.07	1.741 ± 0.08	1.665 ± 0.05	1.679 ± 0.05	NS
LDL (mmol\L	2.656 ± 0.17	2.671 ± 0.17	2.503 ± 0.11	2.564 ± 0.12	NS
Chol (mmol\L)	4.713 ± 0.21	4.579 ± 0.19	4.529 ± 0.13	4.429 ± 0.12	NS
High-sensitivity CRP (mg\L)	7.859 ± 0.13	5.522 ± 0.65	5.852 ± 0.83 (3.39–5.69)	5.71 ± 0.647 (3.21–4.17)	*p* < 0.05 ^a,b^
Creatinine (µmol\L)	56.26 ± 2.02212	56.65 ± 2.07	52.93 ± 1.553	54.52 ± 1.75	NS

LDVD = low-dose vitamin D; HDVD = high-dose vitamin D; B = before treatment; A = after treatment; ^a^ LDVD-B vs. LDVD-A. ^b^ HDVD-B vs. HDVD-A. ^c^ LDVD-A vs. HDVD-A.

**Table 3:** Pearson correlation coefficient of 25(OH)D3 with insulin levels, glucose, and HOMA-IR after treatment with low and high doses of vitamin D.

	LDVD (n = 60)	HDVD (n = 60)
Serum Insulin Pearson correlation (r) Significance	−0.083 NS	−0.236 *p* < 0.05
Serum Glucose Pearson correlation(r) Significance	−0.213 NS	−0.421 NS
HOMA-IR Pearson correlation(r) Significance	−0.134 NS	−0.341 *p* < 0.05

## Data Availability

Not applicable.
